# *Candida* species and other yeasts in the oral cavities of type 2 diabetic patients in Cali, Colombia

**Published:** 2013-03-30

**Authors:** Blanca Lynne Suárez, María Inés Álvarez, Matilde de Bernal, Andrés Collazos

**Affiliations:** a Universidad Antonio Nariño, Cúcuta, Colombia, E-mail: lynnesuag@hotmail.com; b Department of Microbiology, Universidad del Valle, Cali, Colombia, E-mail: malvarez@univalle.edu.co; c Department of Internal Medicine, Endocrinology, Universidad del Valle, Cali, Colombia, E-mails: matilde.mizrachi@correounivalle.edu.co

**Keywords:** Type 2 diabetes mellitus, *Candida* sp, candidiasis, *Prototheca*, Colombia

## Abstract

**Objective:**

To determine the prevalence of *Candida* species and to study factors associated to oral cavity colonization in patients with type 2 diabetes mellitus.

**Methods:**

A total of 107 diabetics were classified into controlled and uncontrolled according to glycosylated hemoglobin values. Each patient was assessed for stimulated salivary flow rates, pH, and an oral rinse to search for yeast. The study also determined the state of oral health via Klein and Palmer CPO indexes for permanent dentition, dental plaque by O'Leary, and a periodontal chart.

**Results:**

We found yeasts in 74.8% of the patients. A total of 36 of the 52 subjects with controlled diabetes presented yeasts and 44 in the uncontrolled; no significant differences (*p* = 0.2) were noted among the presence of yeasts and the control of blood glucose. The largest number of isolates corresponded to *C. albicans*, followed by *C. parapsilosis*. Uncontrolled individuals presented a significantly higher percentage of yeast different from *C. albicans *(*p* = 0.049).

**Conclusions:**

We found a high percentage of *Candida* colonization and uncontrolled individuals had greater diversity of species. The wide range of CFU/mL found both in patients with oral candidiasis, as well as in those without it did not permit distinguishing between colonization and disease. We only found association between isolation of yeasts and the low rate of salivary flow.

## Abstract (es)

**Objetivos:**

Determinar la prevalencia de las especies de *Candida* y estudiar factores asociados a la colonización de la cavidad oral en pacientes con diabetes mellitus tipo 2.

**Métodos:**

107 diabéticos se clasificaron en controlados y no controlados, de acuerdo con los valores de hemoglobina glucosilada. A cada paciente se le tomó una muestra de saliva estimulada y un enjuague oral, la primera se utilizó para determinar el pH y la tasa de flujo salival, la segunda para buscar levaduras. Además, se determinó el estado de salud oral mediante los índices CPO-D de Klein y Palmer para dentición permanente, el de placa bacteriana de O Leary y un periodontograma.

**Resultados:**

Se hallaron levaduras en el 74,8% de los pacientes. Un total de 36 de 52 sujetos con diabetes controlada presentaron levaduras y 44 en los no controlados; sin diferencias significativas (*p* = 0.2) entre la presencia de levaduras y el control de la glucemia. El mayor número de aislamientos correspondió a *C. albicans*, seguido de *C. parapsilosis*. Los individuos no controlados presentaron de manera significante un porcentaje más alto de levaduras diferentes a *C. albicans* (*p* = 0.049). Sólo se encontró asociación entre el aislamiento de levaduras y la baja tasa de flujo salival.

**Conclusión:**

Se encontró un alto porcentaje de colonización por *Candida*, y en los individuos no controlados hubo mayor diversidad de especies. El amplio rango de UFC/mL hallada tanto en pacientes con candidiasis oral como sin ella no permitió establecer colonización y enfermedad.

## Introduction

The *Candida* genus presents over 150 species of which 10 are responsible for infections in humans; of these, *C. albicans* is part of its normal microbiota and is isolated in greatest frequency from the oral cavity in human beings. When the balance between the host and the microorganism is altered, *Candida* becomes pathogenous and oral candidiasis is manifested, as it occurs in diverse populations at risk among which there are individuals infected with the human immunodeficiency virus, with nutritional deficiencies, malignancies, or with metabolic disorders like diabetes mellitus (DM)^1^.

The role of *Candida* in the oral cavity of diabetic subjects is a controversial issue. The prevalence of the microorganism in these patients varies from 18-80%^2^, these discrepancies are attributed to the methodology used in the laboratory, the number and characteristics of the subjects, and to the sampling techniques used^3^ ^-^ ^5^.Although *C. albicans* is the most frequent species, others like *C. dubliniensis, C. glabrata, C. tropicalis, or C. krusei* are also involved in colonization and oral candidiasis^6^.

An exhaustive review of the scientific literature revealed a lack of studies on the association between *Candida* and DM in Colombia, which is why it was considered important to carry out an investigation on *Candida* in the oral cavities of these patients and study some factors that could facilitate the increase of the microorganism and the relationship the pathogen has with oral health.

## Materials and Methods

This cross-sectional prevalence study was conducted on a sample of 107 outpatients with prior diagnosis of Type 2 DM under treatment, 40 years of age or above. All the individuals participated voluntarily and signed a consent form approved by Human Ethics Committee of the Faculty of Health at Universidad del Valle. The study excluded patients who were patients, diagnosed with HIV, or with antifungal treatment within 30 days prior to entering the study. Sample size was calculated through the formula for finite populations^7^, bearing in mind an estimated population of 198,710^8^ ^,^ ^9^diabetics for 2005 in Cali, with an expected 37% prevalence of *Candida* isolates in oral cavity, according to Quirino et al.,^10^ for 95% confidence level and 6% error margin. The patients by convenience were recruited from the Fundation Amanecer Apoyo al Diabético (support to diabetics foundation) and from the medical service at Universidad del Valle in Cali, Colombia.

The study population was classified into controlled and uncontrolled groups according to the level of glycosylated hemoglobin (HbA1c); for the first group, values equal to or below 7% were considered; for the latter values above 7%^11^were considered. HbA1c was quantified through the Bayer DCA-2000 method (specific monoclonal antibody methodology for the A1c fraction).

The soft tissue of the oral cavity of each patient was examined in detail with a dental mirror, recording the possible presence of lesions suggesting candidiasis, which were confirmed by scraping the lesion to perform KOH and culture in CHROMagar *Candida*. Additionally, a blood sample was taken along with samples of stimulated saliva and oral rinse. The first permitted HbA1c measurements, the second was used to determine pH and the stimulated salivary flow rate (SSFR), and the last was used to search for yeasts. The samples were taken between 07:00-10:00 hrs.

To collect the stimulated saliva and oral rinse samples, patients had to be fasting. In addition, if they had removable dentures, they were asked to remove them prior to taking the samples to avoid sweeping the microbial plaque and inert wastes that adhere to denture surfaces.

Before obtaining the specimen of stimulated saliva, patients were asked to swallow to clean the mouth of accumulated saliva and then chew on a piece of flavorless paraffin wax for 5 min and deposit the saliva in a millimetered sterile container^12^. The SSFR calculation was obtained by dividing the volume gathered in the container by five to express it in ml/min; a value below 1 ml/min was considered as hyposalivation and greater than 1 ml/min as normal^12^. The pH was determined as soon as the sample was collected with a pH meter (Pocket-Sized pH meter, Hanna Instruments); values between 6.7 and 7.4 were considered normal, below 6.7 were taken as base and above 7.4 as alkaline^13^.

Subjective xerostomia was determined for each patient by applying FOX criteria^12^. Subjects responding affirmatively to one or more questions from the questionnaire were classified as patients with subjective xerostomia; this data was correlated with the SSFR.

The oral rinse from each patient was obtained with 10 mL of saline solution retained for 30 seconds, then deposited into a sterile container that was stored in a portable cooler at 4 °C, and transported to the mycology laboratory in the Faculty of Health at Universidad del Valle where it was immediately processed. Each sample was homogenized in a vortex, inoculated in CHROMagar *Candida* (CHROMagar Company, Paris, France), and incubated at 30 °C for 48 h. Thereafter, the colony forming units were counted (CFU) per mL. Preliminary identification of yeasts and *Candida* species was done by observing growth in the CHROMagar *candida* ^4^.

The yeast strains presenting colors different from green tonalities in the CHROMagar *Candida* were subjected to API 20C Aux identification (bioMérieux; Marcy-l'Etoile, France). Green colonies presumptive of *C .albicans*/*C. dubliniensis* were subjected to different phenotypic tests that included germ tube formation and production of chlamydospores in Corn Meal Agar^14^. Studies were also made of growth at 42 and 45 °C and in Sabouraud agar by adding sodium chloride at 6.5%^14^. Strains like *C. dubliniensis* were identified with these procedures and confirmed with API 20C Aux.

The state of oral health of the patients was measured through the decayed-missing-filled (DMF) Index by Klein and Palmer^15^ for permanent teeth, O Leary s bacterial plaque index, and a periodontal chart. The first evaluated the state of permanent teeth; the plaque index measured the degree of oral hygiene of each of the patients (a value of bacterial plaque above 20% indicated inadequate oral hygiene).

The periodontal chart was made to assess the periodontal state and evaluate treatment needs, and the community periodontal index of treatment needs (CPITN)^16^ was used for this purpose. 

The results were included in a database and their analysis was made via the Epi-info 6.04 statistical program. To measure the association among the different variables, the Chi square test was used; when a relationship existed, the Odds Ratio was determined.

## Results

Of the 107 patients studied, 52 were classified as controlled and 55 as uncontrolled. Age ranged between 42 and 83 years with a mean of 62 years. Yeasts were present in 69.2% of the patients with controlled DM (36/52) and in 80% of those uncontrolled (44/55), without significant statistical differences between the presence of yeasts and glycemia control (*p*= 0.2).

The prevalence of *Candida* in the population studied was 71% (76/107) and the genuses different from *candida* 16.8% (18/107) corresponded to *Rhodotorula, Trichosporon, Saccharomyces, Cryptococcus, Kloeckera,* and the *Prototheca algae* (Table 1); this was identified at genus level through microscopic observation of teaks and the species via API 20C Aux.

**Table 1:**
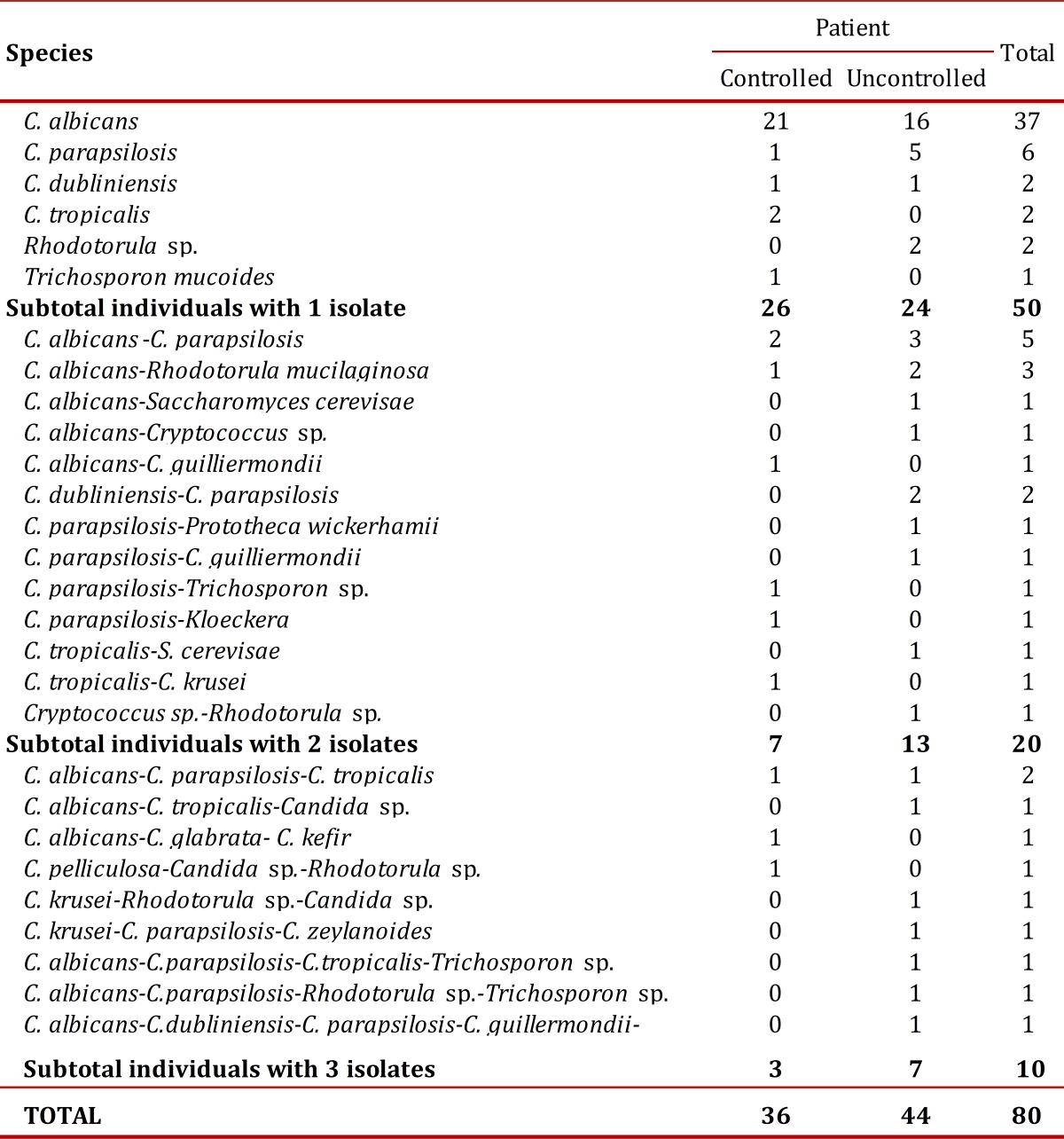
Species of yeasts and algae isolated in both groups of diabetic patients

The results of the distribution of *C. albicans* and of the yeasts differing from *C. albicans* showed that, of the 37 patients with *C. albicans*, 21 were control patients and 16 were non-control patients, while with the 43 patients with yeasts differing from *C. albicans*, 15 were control individuals and 28 were non-control patients. This difference was statistically significant (*p*= 0.049)

The subjects colonized by yeasts were classified according to the CFU count in three groups: 45 (56.2%) subjects were found in the first group of ≤50 CFU/mL, 24 (30%) subjects had 51-400 CFU/ml, and 11 (13.8%) had a count >400 CFU/mL. The minimum CFU value was 1.25 and the maximum was 20.450 CFU/mL, with this last figure being completely atypical and it was eliminated to avoid bad interpretation of the results. With this figure excluded, the maximum value was 3,397.5 CFU/mL and the average was 182.2 CFU/mL. No statistical association was found among the number of CFU/mL of yeasts and glycemia control, the state of oral health, pH or the use of removable dentures.

Table 2 presents the relationship between the presence of yeasts and the different characteristics found in the oral cavities of the patients studied. Statistically significant association was found with low SSFR (OR 2.448, 95% CI 0.995 - 6.023) and with the presence of removable dentures (OR 2.903. 95% CI 1.138 - 7.402).

**Table 2.**
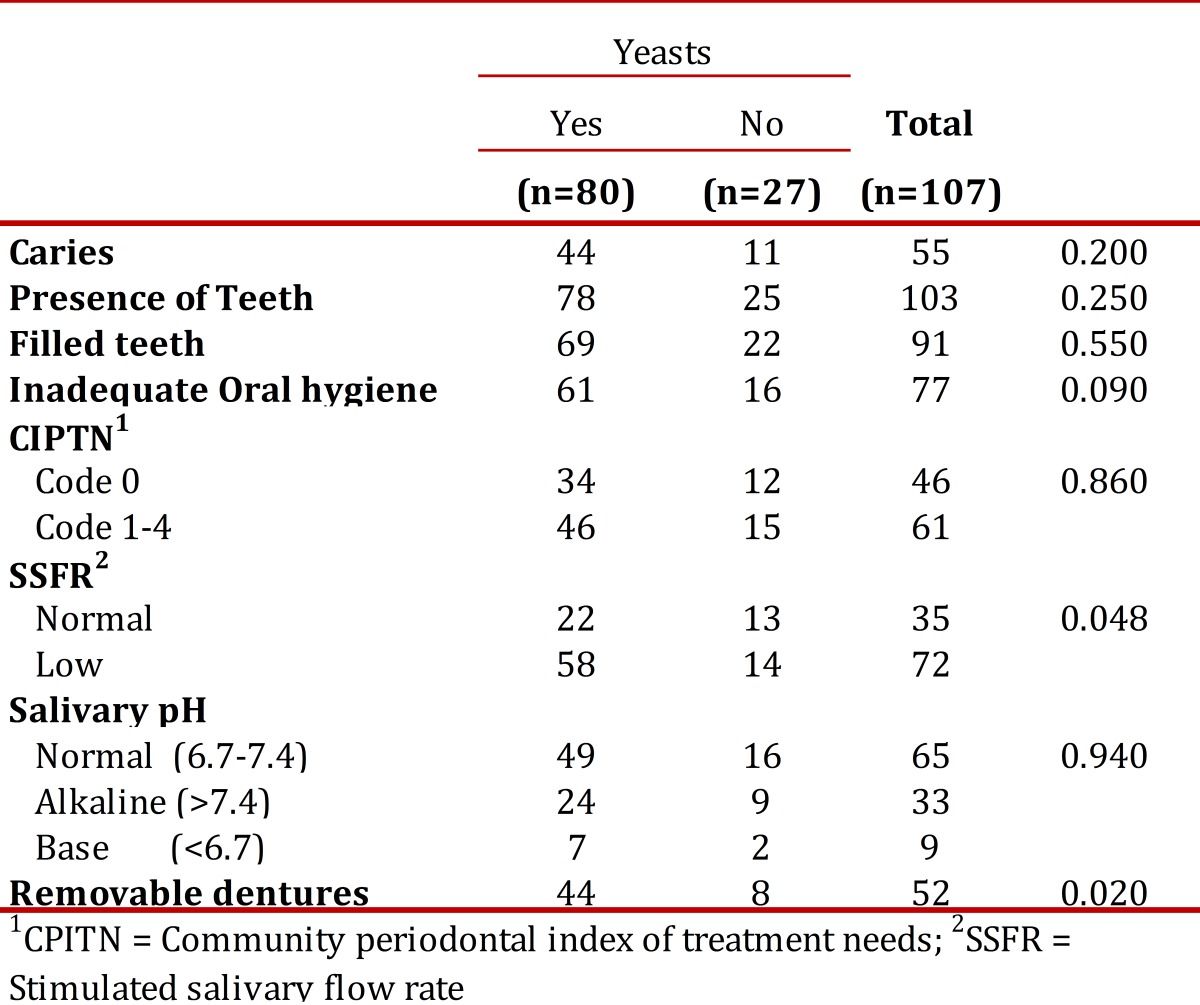
Characteristics of the oral cavity and presence of yeasts in the patients studied.

Information on subjective xerostomia was obtained from 106 individuals; one was excluded due to mental disability to report this symptom. Of the 64 (60.4%) subjects with xerostomia, 73.4% (47/64) had diminished SSFR. In the 42 (39.6%) individuals who did not report sensation of xerostomia, 57.1% (24/42) had hyposalivation without statistically significant differences (*p* = 0.081). Twelve patients (11.2% of the 107 studied) presented oral candidiasis (Table 3), all with erythematous candidiasis; six of them had removable dentures. The CFU/ml count of yeasts was between 2.5 and 3183.75; only two subjects presented counts above 400 CFU/ml, one corresponding to the controlled group (427.5 CFU/ml) and the other to the uncontrolled group (3183.75 CFU/mL). These data did not permit establishing a cutoff point to differentiate between colonization and clinical infection.

**Table 3:**
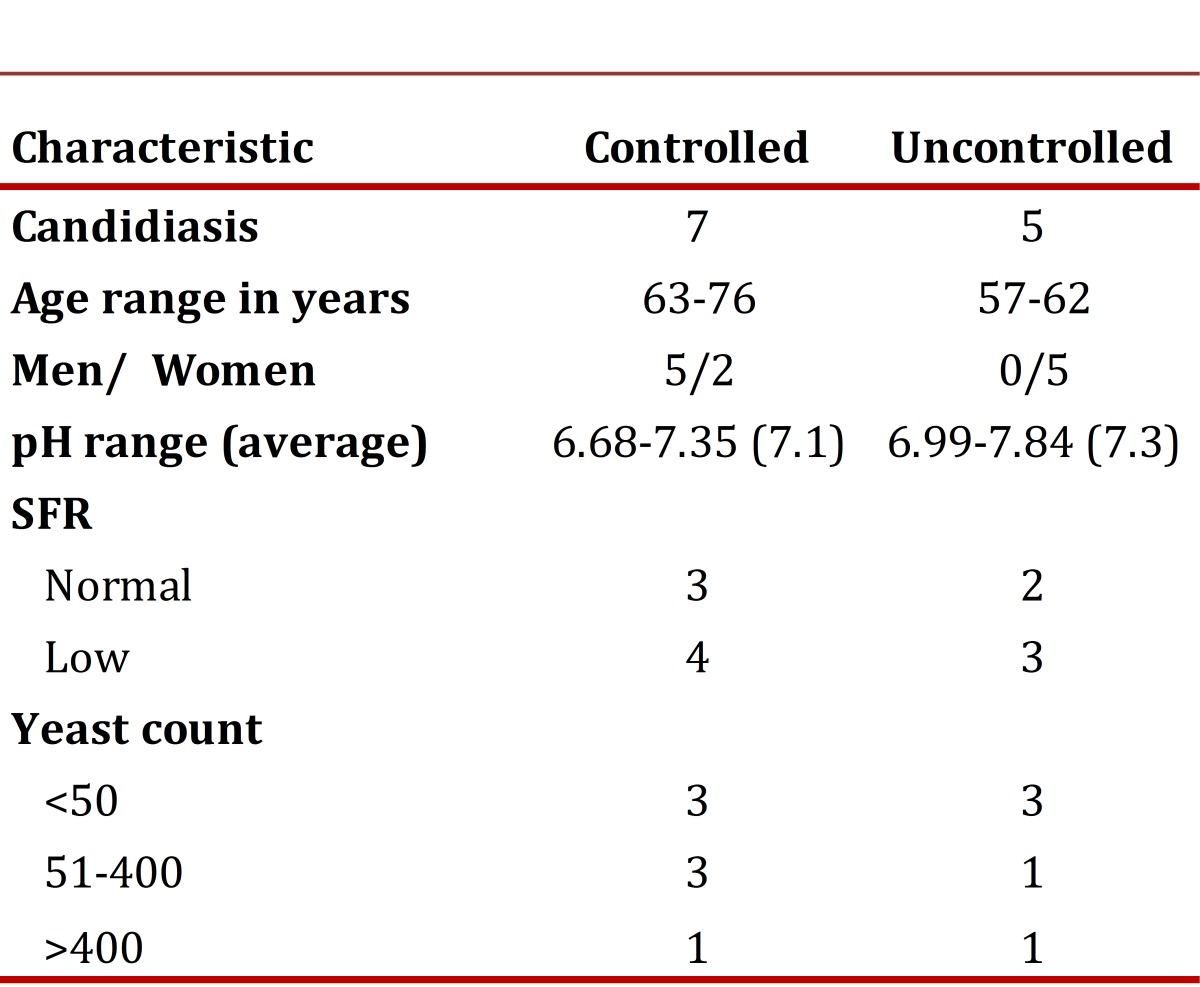
Characteristics of controlled and uncontrolled diabetic patients with oral candidiasis

The HbA1c average among subjects with oral candidiasis was 6.9%, while that of the individuals without oral candidiasis was 7.4%, without finding statistical differences between the averages (*p*= 0.3121 Two-sampled Independent t Test). 

 Of the 12 individuals with candidiasis, in nine (75%) *C. albicans* was isolated and in one of them *C. dubliniensis* was also found.Of the three remaining without *C. albicans* isolates, two (17%) were found with *C. parapsilosis* and one (8%) with *C. tropicalis*.

## Discussion

The presence of yeasts in the oral cavities of diabetic patients has been researched in several parts of the world, with quite diverse values^2^ ^,^ ^4^. The prevalence of yeasts obtained in our sample (from centers with experience in caring for diabetic patients) was 74.8%, confirming that variability, which could depend on social factors and on the sampling technique.

Khosravi* et al*.,^6^mention that the degree of yeast colonization of the oral cavity may be altered by levels of blood glucose; however, our study did not reveal statistically significant differences between controlled and uncontrolled individuals. This data agrees with the report by *Manfredi et al*.,^5^ who state that the frequency of isolates and the carrier state of *Candida* is independent of the HbA1c level, and suggest that growth of yeasts in the oral cavities of diabetic patients depends on other factors. It has been reported that a level of HbA1c above 12% is highly predictive of oral infection due to *candida* ^2^; however, our study with 107 diabetics only found two individuals with values of HbA1c above 12% (only one of them with presence of *C albicans*); that low number of individuals with HbA1c at that level does not permit drawing conclusions.

Different works^1^ ^,^ ^3^ ^,^ ^4^ ^,^ ^6^ indicate *C. albicans* as the isolated yeast with the highest frequency in the oral cavities of diabetic patients. In this research, this species was the most prevalent and it was found more frequently in controlled subjects, while in uncontrolled subjects, isolates from other yeasts predominated with statistically significant differences. Other studies with diabetic patients and healthy controls found that the species of C. not albicans were more prevalent in diabetic individuals^3^ ^,^ ^4^ ^,^ ^6^. It is likely that these results indicate that glucose concentration, in uncontrolled diabetics, keeps relation with the finding of yeasts different from *C. albicans* in the oral cavities of these subjects. Something similar has been observed in patients with HIV, where isolates of species of C. not albicans is more prevalent in advanced stages of the disease^17^. The species of C. not albicans have been related to the resistance to antifungal substances^17^; in this work, such factor cannot be related to the presence of these yeasts, given that we excluded patients in antifungal therapy and no tests were conducted on susceptibility to these medications.

It is highlighted that this research developed the first isolates of *C. dubliniensis* in Colombia, which was previously published^14^. This is emergent pathogenous yeast isolated mainly in the oral cavities of patients infected with HIV^17^. Additionally, two uncontrolled patients were found with Prototheca wickerhamii algae, which is common in nature and it is known that it causes localized or disseminated infection in humans, both in immunocompetent and immunocompromised patients^18^. The national and international literature review did not find reports of Prototheca in the oral cavities of diabetic subjects and its role in this site is unknown.

In a study with type 2 diabetic patients, Hintao *et al*.,^19^ reported that yeasts, especially *C. albicans*, are related to coronal and root caries. This research did not find association between the DMF index and the presence of yeasts. 

Diabetes Mellitus is considered a risk factor for the development of periodontal disease, above all in patients with poor control of the disease^2^. This study did not find statistical association between CPITN and glycemic control. Codes 3 and 4, which reflect greater periodontal severity, were very similar for both controlled and uncontrolled diabetic patients. These results coincide with the study carried out by Arrieta *et al*.,^16^ in type 1 and 2 diabetic patients, which reported that no relationship existed between CPITN and metabolic control of the disease.

Regarding bacterial plaque index and yeast isolates, the current study found that of 80 patients with yeasts, 61 (76.3%) presented inadequate oral hygiene, but these data did not show significant differences (Table 2). In this case, the degree of accumulation of bacterial plaque may be secondary to other factors.

Studies conducted in vitro describe that a correlation exists among *Candida* growth, acid production, and proteolysis; given that yeast cultures in saliva reveal stimulated growth due to diminished pH^20^. This study did not find association among the different levels of salivary pH and glycemic control of the disease, or with the presence of yeasts; inclusively, in the 12 patients with oral candidiasis, 10 had pH within the normal range. However, Kadir *et al*.,^3^ reported lower pH values in diabetic patients when compared with non-diabetic individuals. In the current research, the salivary pH variations were independent of glycemic control and it was not a factor that would affect *Candida* growth.

Moore *et al*.,^21^ affirm that xerostomia is not necessarily associated to diminished amounts of saliva and described, in type 1 diabetic patients, a significant relationship among hyposalivation, poor glycemic control, and medications that produce xerostomia. Additionally, they reported that high levels of HbA1c associated to hyposalivation can indicate progression of the disease.

This research did not find association between subjective xerostomia and SSFR; the latter showed significant association with the presence of yeasts, which points to hyposalivation as a factor predisposing to greater colonization by these microorganisms possibly because of diminished elements in saliva with antifungal properties, as well as because of the sweeping action and immunological protection provided by this liquid^22^.In this work, patients with removable dentures presented a higher presence of yeasts (*p*= 0.02); similar results have been reported in previous studies in individuals with DM^3^ ^,^ ^6^.

This study noted chronic erythematous candidiasis in 12 individuals; however, CFU counts of these patients were quite variable and did not permit establishing a cutoff point, given that subjects with candidiasis and low counts were found, as well as individuals without candidiasis with high and low CFU counts; results similar to those reported by other researchers^2^. Although no association was seen between the level of HbA1c and the presence or not of chronic erythematous candidiasis, the very low number of subjects does not permit generalizing that observation. Sousa *et al*.,^23^ in a study conducted with healthy subjects and type 2 diabetic patients concluded that no relationship was found between the level of glycemia and the presence of oral candidiasis; however, the method to determine glycemic control was the measurement of capillary glucose through glucometer and not through the level of HbA1c. Hence, it is necessary to conduct a study comparing glycemic control by measuring HbA1c in both groups to obtain a valid conclusion in patients with oral infection due to *Candida*.

The finding of three cases of oral candidiasis without *C. albicans* isolates (two with *C. parapsilosis* and one with *C. tropicalis*) corroborates other reports that highlight the importance of the non- albicans *Candida* species as pathogens in humans. *C. parapsilosis* has emerged as a pathogen in systemic infections in recent decades, identifying the following risk factors: immunocompromise (patients with AIDS, neutropenic patients), with prolonged use of intravascular devices or prosthetic material^24^; however, reports as responsible for oral candidiasis are scarce^25^ or it has not been systemically sought in some studies.

The course of oral candidiasis in diabetics due to *C. parapsilosis*, as well as the response to antifungal treatment is still unknown. The diversity of isolated yeasts in the oral cavities of diabetic subjects and the association between poor control of diabetes and the species of *C. non-albicans*, which generally present little sensitivity to some antifungal substances, creates the need for new studies that permit to more clearly establish the role of *Candida* in the oral cavities of type 2 diabetic patients.
